# Molecular Sexing in Owls (Aves, Strigiformes) and the Unique Genetic Structure of the Chromodomain Helicase DNA-Binding Protein 1 (CHD1) Gene on Chromosome W

**DOI:** 10.3390/genes16060653

**Published:** 2025-05-28

**Authors:** Mana Esaki, Kenki Momohara, Atsushi Haga, Maria Narahashi, Mu Mu Aung, Kaori Tokorozaki, Yuko Haraguchi, Kosuke Okuya, Isao Nishiumi, Manabu Onuma, Makoto Ozawa

**Affiliations:** 1 Joint Graduate School of Veterinary Science, Kagoshima University, Kagoshima 890-0065, Japan; k0068103@kadai.jp (M.E.); kokuya@vet.kagoshima-u.ac.jp (K.O.); 2 Department of Pathogenetic and Preventive Veterinary Science, Joint Faculty of Veterinary Medicine, Kagoshima University, Kagoshima 890-0065, Japan; k0828113@kadai.jp; 3 National Institute for Environmental Studies, Tsukuba 305-8506, Japan; haga.atsushi@wbnw.jp (A.H.); monuma@nies.go.jp (M.O.); 4 Graduate School of Integrated Sciences for Global Society, Kyushu University, Fukuoka 819-0395, Japan; narahashi.maria.392@s.kyushu-u.ac.jp; 5 Forest Research Institute, Forest Department, Ministry of Natural Resources and Environmental Conservation, Yezin, Nay Pyi Taw P.O. Box 05282, Myanmar; mumuaung85@gmail.com; 6 Kagoshima Crane Conservancy, Izumi 899-0208, Japan; anag0mama@po2.synapse.ne.jp (K.T.); crane_c@city.izumi.kagoshima.jp (Y.H.); 7 Transboundary Animal Diseases Research Center, Joint Faculty of Veterinary Medicine, Kagoshima University, Kagoshima 890-0065, Japan; 8 Department of Zoology, National Museum of Nature and Science Tokyo, Tsukuba 305-0005, Japan; nishiumi@kahaku.go.jp

**Keywords:** molecular sexing, *CHD1* gene, owl

## Abstract

Background: The accurate determination of bird sex is crucial in various biological fields, including ecology, behavioral research, and conservation. However, this task remains challenging in species in which males and females exhibit similar external morphologies, such as owls. Although polymerase chain reaction (PCR)-based molecular sexing techniques that target the chromodomain helicase DNA-binding protein 1 gene found on sex chromosomes Z (*CHD1-Z* gene) and W (*CHD1-W* gene) are widely used, we encountered atypical banding patterns when applying the previously reported primers 2550F and 2718R to four wild owls of unknown sex. This study aims to reveal the owl-specific genetic structure of the *CHD1* gene. Methods: We developed a new primer set and determined the nucleotide sequences—including the binding sites for the primers 2550F and 2718R—within both the *CHD1-Z* and *CHD1-W* genes. Results: Sequencing analysis, conducted using a newly developed primer set that successfully amplified both Z- and W-derived *CHD1* products across various owl species, revealed a unique genetic insertion of approximately 600 bp in intron 17 of the *CHD1-W* gene. This insertion reversed the usual length relationship between PCR products from the chromosomes Z and W. Additionally, mutations identified in the 2550F primer binding site of the *CHD1-Z* gene in certain owl species may explain the failure to amplify *CHD1-Z*-derived PCR products. Conclusion: These findings provide valuable insights for improving molecular sexing in owls.

## 1. Introduction

The precise determination of a bird’s sex is essential in various biological fields, including ecology, behavioral research, and conservation [1,2]. However, distinguishing between males and females based solely on external morphology can be challenging in monomorphic species [3,4,5].

Molecular sexing techniques using polymerase chain reaction (PCR) have been widely adopted owing to their accuracy and speed. Birds possess two sex chromosomes, Z and W. Males are homogametic (ZZ), and females are heterogametic (ZW) [6]. DNA-based sex identification in birds exploits the differences in nucleotide sequences between these chromosomes. Specifically, the chromodomain helicase DNA-binding protein 1 (*CHD1*) gene, found on both chromosomes, differs in intron length between the Z and W alleles [2,7,8]. By amplifying these regions with specific primers, females typically display the two distinct bands on agarose gels: a longer band from chromosome Z and a shorter band from chromosome W. In contrast, males show a single band corresponding to two copies of chromosome Z [9].

In this study, we initially applied the conventional molecular sexing method on four wild owls of unknown sex collected in Japan, including *Asio otus* (long-eared owl), *Otus semitorques* (Japanese scops owl), *Strix uralensis hondoensis* (Ural owl), and *Ninox japonica* (northern boobook), but observed atypical band lengths for the PCR products. To understand these unusual molecular sexing results, we aimed to reveal the owl-specific genetic structure of the *CHD1* gene, including primer binding sites, by determining the nucleotide sequences of the PCR products from these four owls, as well as those from other owl species using a newly designed primer set.

## 2. Materials and Methods

### 2.1. Sample Collection

We analyzed DNA extracted from 23 samples, including muscle, liver, cultured cells, and swabs from various owl species and *Grus monacha* (hooded crane) (Table 1). Twelve and six owl samples were collected at the National Institute for Environmental Studies (NIES) and the National Museum of Nature and Science (NMNS), respectively. All owl samples, except those from *Otus lettia* (Collared scops owl), *Tyto alba* (barn owl), and *Tyto longimembris* (Eastern grass owl), were collected from individuals residing in Japan. Specimens of *O. lettia*, *T. alba*, and *T. longimembris* were collected in Myanmar. Samples from an *Asio flammeus* (short-eared owl) and two *G. monacha* were collected on the Izumi plain, Kagoshima prefecture, Japan, and processed at Kagoshima University. Additionally, one sample each from *A. otus* and *O. semitorques* was collected from individuals rescued and reared at Kagoshima University. The samples collected by the National Museum of Nature and Science were obtained between August 2022 and March 2023. Of the 23 birds analyzed in this study, 11 deceased birds were sexed by necropsy through dissection of their reproductive organs (Table 1). DNA was extracted from the samples using the innuPREP Virus DNA/RNA Kit (Analytik Jena AG, Jena, Germany), as described previously [10].

### 2.2. PCR Amplification of the CHD1 and COI Genes

**Figure 1:**
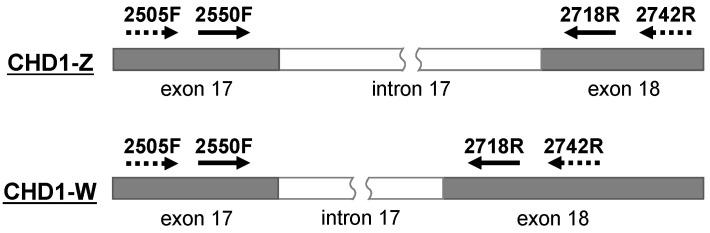
Primers used for molecular sexing in this study. The previously reported primers 2550F and 2718R [13] target conserved nucleotide sequences in exons 17 and 18, respectively, of *CHD1* in both the *CHD1-Z* and *CHD1-W* genes. New primers, 2505F and 2742R, were designed based on the chromosomal DNA sequences of the *CHD1-Z* and *CHD1-W* genes.

Partial regions of the chromosomal *CHD1* and mitochondrial *COI* genes were amplified from nucleic acids extracted from the samples using Tks Gflex DNA polymerase (TaKaRa Bio, Otsu, Japan) and the primers described in Figure 1 and Table 2. The previously reported primers 2550F and 2718R [11], which target conserved nucleotide sequences in exons 17 and 18, respectively, of the *CHD1* gene on both chromosomes Z (*CHD1-Z*) and W (*CHD1-W*), are known for their utility in differentiating sexes in birds by amplifying the distinct lengths of intron 17 flanked by these exons. This primer set has been extensively used in various avian sexing studies [7,8,12,13,14,15,16,17,18].

Primers 2505F and 2742R (Table 2) were newly designed for this study based on conserved *CHD1-Z* and *CHD1-W* gene sequences from species including two Accipitridae: *Aquila chrysaetos chrysaetos* (Z: accession no. LR606180.1; W: accession no. HG999777.1), *Accipiter gentilis* (Z: OV839360.1, W: OV839371.1); one Columbidae: *Streptopelia turtur* (Z: LR594555.2, W: OU015480.1); one Caprimulgidae: *Caprimulgus europaeus* (Z: OU015527.1, W: OU015537.1); one Acrocephalidae: *Acrocephalus scirpaceus scirpaceus* (W: OU383795.1); one Muscicapidae: *Erithacus rubecula* (W: LR812130.2); and one Phasianidae: *Gallus gallus* (W: AC177807.2). These primers allow for the confirmation of the primer binding sites targeted by 2550F and 2718R.

Additionally, the mitochondrial cytochrome *c* oxidase I (*COI*) gene, which is crucial for species identification, was amplified using the well-documented primers Bird F1 and Bird R1 [19], in order to phylogenetically infer the origin of the amplified *CHD1* gene. We amplified each gene using the Tks Gflex DNA Polymerase. PCR products were loaded onto a 2% agarose gel for electrophoresis and visualized using ethidium bromide under ultraviolet light.

Each 20 µL PCR reaction mixture consisted of 10 µL of 2× Gflex Buffer, 0.4 µL of Tks Gflex polymerase, 7.4 µL of nuclease-free water, 0.6 µL of each relevant primer (10 µM), and 1 µL of extracted nucleic acids. The PCR conditions included an incubation at 94 °C for 1 min followed by 40 cycles of denaturation at 98 °C for 10 s, annealing at 55 °C for 15 s, and extension at 68 °C for 1 min, with a final extension at 68 °C for 5 min. PCR products were loaded onto a 2% agarose gel for electrophoresis and visualized using ethidium bromide under ultraviolet light.

### 2.3. DNA Sequencing

The PCR products were purified using the Wizard SV Gel and PCR Clean-Up System (Promega Corporation, Madison, WI, USA). The DNA sequences of the purified PCR products were determined using Sanger sequencing at Azenta Life Sciences (Tokyo, Japan) to identify the origin of the amplified fragments. The DNA sequences were aligned using MEGA software version X [20], together with the corresponding sequences of the *CHD1-Z* and *CHD1-W* genes of 36 to 38 bird species (Supplementary Table S1) deposited in the NCBI GenBank database (https://www.ncbi.nlm.nih.gov/genbank/, accessed on 2 September 2024). Specifically, we determined the chromosomal DNA sequences corresponding to the partial exon 17 sequence, the full-length intron 17 sequence, and the partial exon 18 sequence of *CHD1-Z* and *CHD1-W* in 14 bird species. These sequences were deposited in the GenBank database under the accession numbers listed in Table 3.

### 2.4. Phylogenetic Analysis

The DNA sequences of the chromosomal *CHD1* and mitochondrial *COI* genes determined in this study were phylogenetically analyzed alongside the corresponding sequences from representative bird species (Supplementary Table S1) retrieved from the GenBank database, in order to verify the chromosomal origin of the *CHD1*-derived PCR products obtained from owls in this study. Phylogenetic trees for each gene were constructed using MEGA software version 7 [21] with the maximum likelihood method, supported by 1000 bootstrap replicates to assess the robustness of the inferred nodes.

### 2.5. Ethics Statement

This study was conducted in compliance with the International Guiding Principles for Biomedical Research Involving Animals, the Japanese Law for Conservation of Endangered Species of Wild Fauna and Flora, and the regulations of the Kagoshima University Research Ethics Committee. No animal experiments were conducted as part of this study. Swab samples from long-eared and Japanese scops owls were collected by veterinary staff at Kagoshima University primarily for diagnostic purposes. All handling procedures were carried out with the aim of minimizing stress and ensuring the welfare of the subjects.

## 3. Results and Discussion

### 3.1. Molecular Sexing of Four Wild Owls of Unknown Sex

It is highly challenging to distinguish between the males and females of some bird species, including owls, based on external morphological characteristics. Commonly, PCR-based techniques have been widely employed for sex determination in bird species by targeting the *CHD1* gene, which is found on the sex chromosomes Z and W [12,15,22,23]. First, we aimed to determine the sex of four individual wild owls of unknown sex using conventional molecular sexing methods with the previously reported primer set, 2550F/2718R [11]. The four owl species tested included the *A. otus*, *O. semitorques*, *S. uralensis hondoensis* (Ural owl), and *N. japonica* (northern boobook) (Table 1). Male and female *G. monacha* served as reference samples for typical avian molecular sexing patterns (Table 1).

The male *G. monacha* displayed a single band of approximately 600 bp, whereas female *G. monacha* showed two bands of approximately 400 and 600 bp, in line with our expectations (Supplementary Figure S1). The band patterns of the four owl species differed from those of the cranes. The *A. otus* and *O. semitorques* showed a single band of approximately 1 k bp. Additionally, the *S. uralensis hondoensis* and *N. japonica* exhibited two bands, one of approximately 1k bp and the other approximately 600 bp.

To ascertain the origin of these PCR products, we sequenced each band and conducted basic local alignment search tool (BLAST) searches against the NCBI GenBank database. The 600 bp PCR products from the *S. uralensis hondoensis* and the *N. japonica* matched (92.65–99.33% similarity) the *CHD1-Z* genes of other owl species, including the *Strix nebulosa* (great grey owl) (accession no. KF601354.1) and *A. flammeus* (accession no. KF601360.1). These results confirmed their derivation from chromosome Z. Conversely, the 1k bp PCR products from all four owl species showed high similarity (93.26–94.73%) to the *CHD* gene of the *Megascops asio* (eastern screech owl) (accession no. HQ593874.1), the chromosomal origin of which remained unclassified in the database. These findings suggested that the 1k bp PCR products likely originated from chromosome W, indicating a significant divergence in the *CHD1-W* gene structure in owls compared to other birds. All four wild owls were identified as females, each possessing chromosome W.

### 3.2. Application of a New Primer Set 2505F/2742R in Owls

The results described above also highlight the potential limitations of the previously reported primer set 2550F/2718R for amplifying *CHD1-Z* gene targets from *A. otus* and *O. semitorques*. To determine the nucleotide sequences—including the binding sites for the primers 2550F and 2718R—within both the *CHD1-Z* and *CHD1-W* genes, we developed a new primer set, 2505F/2742R. This new primer set was designed to bind outside the regions targeted by the previously reported primer set 2550F/2718R (Figure 1), based on analysis of eight *CHD1* gene sequences from four bird species available in the GenBank database. Using the new primer set, we successfully amplified two distinct bands of approximately 600 and 1 k bp from both *A. otus* and *O. semitorques*.

**Figure 2:**
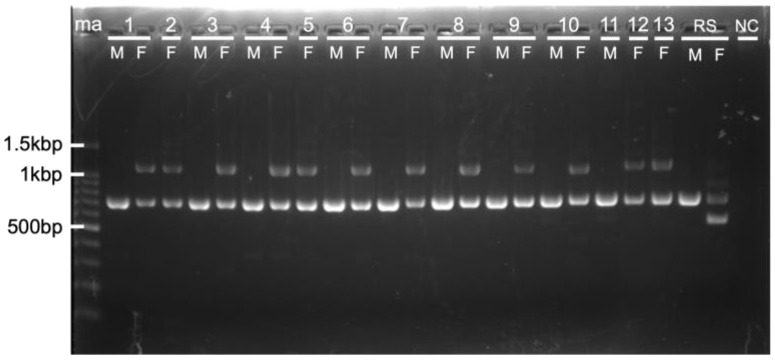
Agarose gel electrophoretic image showing PCR products amplified using the primer set 2505F/2742R. The new primer set, 2505F/2742R, was used to analyze 21 samples, including 9 males (M) and 12 females (F) from 13 owl species. A *G. monacha* (hooded crane) sample was included as a reference sample (RS), and nuclease-free water was used as a negative control (NC). 1: *A. otus* (long-eared owl), 2: *A. flammeus* (short-eared owl), 3: *S. uralensis hondoensis* (Ural owl), 4: *S. uralensis japonica* (Yezo Ural owl), 5: *Otus sunia* (oriental scops owl), 6: *O. elegans interpositus* (Ryukyu scops owl), 7: *O. semitorques* (Japanese scops owl), 8: *O. lettia* (collared scops owl), 9: *N. japonica* (northern boobook), 10: *A. brama* (spotted owlet), 11: *B. bubo* (Eurasian eagle-owl), 12: *T. alba* (barn owl), 13: *T. longimembris* (eastern grass owl), ma: 100-bp DNA ladder used as a marker.

To investigate whether the new primer set was applicable to molecular sexing across a broad range of owl species, 21 individuals from 13 owl species (Table 1) were tested. Owls (Strigiformes) are divided into two families: Tytonidae (barn owls), comprising 19 species, and Strigidae (typical owls), comprising 194 species [24,25]. Among the 13 owl species, two belong to the family Tytonidae and the remaining 11 to Strigidae. While 9 male owls produced a single band of approximately 600 bp, 12 female owls produced two bands, one of approximately 1k bp and the other of approximately 600 bp (Figure 2). The male and female cranes also showed single and double bands, respectively (Figure 2), mirroring the results obtained using the previously reported primer set 2550F/2718R (Supplementary Figure S1). Notably, when using the previously reported primer set 2550F/2718R, the shorter bands typically observed were absent in three owl species: female *A. otus*, female *O. semitorques*, and female *A. flammeus* (Supplementary Figure S2). These results highlight the limited reliability of the primer set 2550F/2718R for molecular sexing in these three owl species.

### 3.3. Confirmation of the Origin of the CHD1 Gene with Phylogenetic Analyses

**Figure 3:**
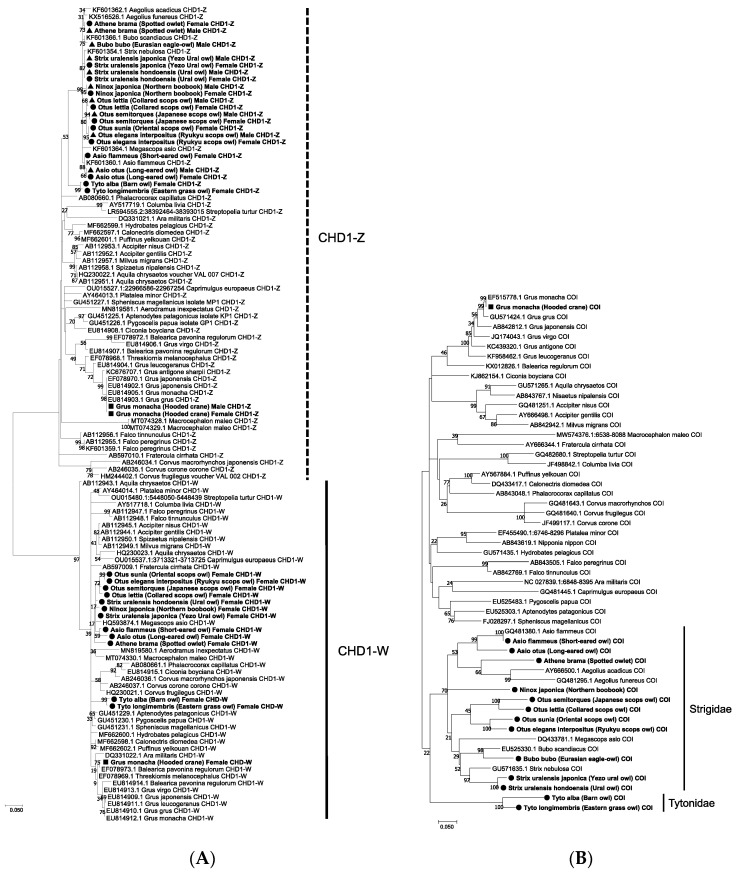
Phylogenetic trees of the *CHD1* and *COI* genes from representative bird species. Twenty-nine nucleotide sequences of *CHD1* (**A**) and 14 nucleotide sequences of *COI* (**B**) determined in this study were phylogenetically aligned with corresponding sequences from representative bird species deposited in the GenBank database. Circles denote sequences from females, triangles denote sequences from males, and squares denote sequences from reference samples that were sequenced in this study. Phylogenetic trees were constructed using the maximum likelihood method with 1000 bootstrap replicates. The scale bar indicates the number of nucleotide substitutions per site.

To verify the chromosomal origin of the *CHD1*-derived PCR products obtained from owls in this study, phylogenetic analyses were conducted using the *CHD1-Z* and *CHD1-W* genes from various avian species available in the GenBank database. The analysis showed that the short PCR product of approximately 600 bp from owls clustered with the *CHD1-Z* genes of other bird species, whereas the long PCR product greater than 1k bp clustered with *CHD1-W* genes (Figure 3A). This suggests that in owls, the 1k bp PCR products are derived from *CHD1-W*, and unlike in other birds, the long-short relationship between *CHD1-Z* and *CHD1-W* is reversed.

Additionally, phylogenetic analysis indicated that the two owl species from Tytonidae tended to cluster separately from those of Strigidae, which was consistent with the results of the *COI*-based phylogenetic analysis (Figure 3B). This supports the notion of genetic divergence between Strigidae and Tytonidae, highlighting unique evolutionary paths within the owl lineage.

### 3.4. Alignment of CHD1 Genes in Owls

**Figure 4:**
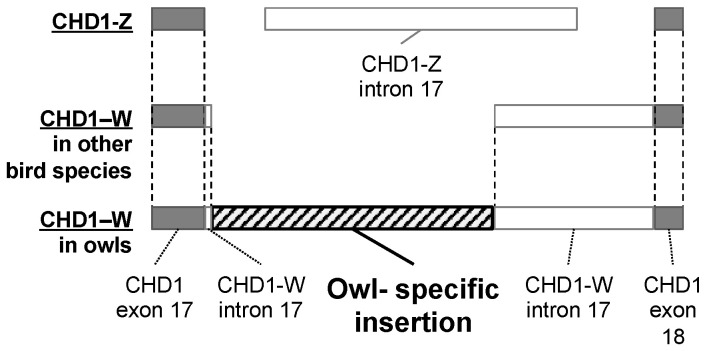
Schematic representation of PCR products from the *CHD1-Z* and *CHD1-W* genes amplified using the primer set 2550F/2718R. In both *CHD1-Z* and *CHD1-W*, intron 17 (white bars) was flanked by exons 17 and 18 (gray bars). These intronic regions begin with GT and end with AG, following the GT-AG rule of introns. An owl-specific region of approximately 600 bp (striped bar) was inserted into intron 17 of *CHD1-W*.

To determine the reason for the elongation of *CHD1-W* genes in owls compared to those in other bird species, we aligned *CHD1* gene sequences from various owl species with those from other birds and analyzed their genetic composition. All PCR products consisted of exon 17, intron 17, and exon 18 of the *CHD1* genes, with the exon-intron boundaries conforming to the GT-AG rule. In owls, we discovered a specific insertion of approximately 600 bp in intron 17 of *CHD1-W* (Figure 4). This insertion likely occurred during the phylogenetic evolution of owls, contributing to the elongation of *CHD1-W*.

**Figure 5:**
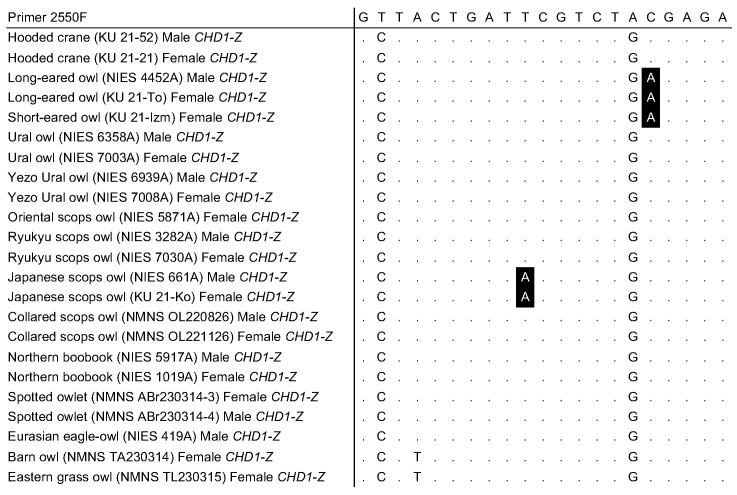
Sequence alignment of the *CHD1-Z* sequence at the binding site for the primer 2550F in various owl species. A dot (.) indicates the same nucleotide as the primer sequence.

Additionally, when examining the binding site for the primer set 2550F/2718R in various owl species, multiple base differences were identified at the 2550F binding site in the *CHD1-Z* gene in three owl species―*A. otus*, *A. flammeus*, and *O. semitorques*―that were not present in the other owl species (Figure 5). This discrepancy suggested that, in females of these three owl species, PCR products derived from *CHD1-Z* were not amplified, leading to the appearance of a single band on gel electrophoresis, as shown in Supplementary Figure S2. At the remaining binding sites for the primer set 2550F/2718R, only minor variations of fewer than two bases compared to the primer sequences were identified (Supplementary Figure S3A,B).

## 4. Discussion

In this study, we elucidated the owl-specific genetic structure of the *CHD1* gene amplified using the primer set 2550F/2718R and clarified the reason for the inversion in PCR product lengths between the *CHD1-Z* and *CHD1-W* genes. Furthermore, using a newly designed primer set, 2505F/2742R, we demonstrated that sequence divergence in the flanking regions of the *CHD1* gene, specifically at the primer binding sites, can prevent amplification of *CHD1-Z* in certain owl species. Although this new primer set enables detailed analysis of the primer binding regions and shows potential for improving the accuracy of molecular sex determination, further investigation is required to confirm its effectiveness.

Through sequencing and alignment of PCR products, we identified a specific insertion within intron 17 that accounts for the distinctly longer *CHD1-W* amplicons observed in owls. This insertion was confirmed in 12 owl species predominantly distributed in Japan. Notably, earlier studies using the 2550F/2718R primer set for molecular sexing of owls reported female-specific PCR products of approximately 1 kb in length [2,11,16,17,18,26,27], supporting the likelihood of a similar insertion in species such as *Ketupa flavipes* (Tawny Fish Owl), *Aegolius funereus* (Tengmalm’s Owl), *Otus scops* (Eurasian Scops Owl), *O. elegans* (Elegant Scops Owl), *Strix leptogrammica* (Brown Wood Owl), and *Otus bakkamoena* (Collared Scops Owl).

Given that molecular sexing typically relies on the interpretation of electrophoretic banding patterns, our findings on the inversion of *CHD1* amplicon sizes in owls may help prevent potential misinterpretations. In three owl species examined in this study, *A. otus*, *A. flammeus*, and *O. semitorques*, the 2550F/2718R primer set failed to amplify *CHD1-Z*-derived PCR products. Sequence analysis suggested that this failure was due to specific nucleotide differences at the 2550F primer binding site of *CHD1-Z*. These differences may account for misidentifications in molecular sexing. Among the *Otus* species examined, such sequence divergence was found only in *O. semitorques*, suggesting that the mutation is species-specific.

Although only male samples of *B. bubo* (Eurasian Eagle-Owl) were analyzed in this study, previous reports have noted the absence of *CHD1-W* amplification in female individuals of this species [12,18,28], indicating the possible presence of base substitutions at the *CHD1-W* primer binding site in *B. bubo*. The new primer set, 2505F/2742R, successfully enabled sequencing of the primer-binding regions targeted by 2550F/2718R and achieved accurate sex determination in owls. Given its demonstrated utility in *G. monacha* (hooded crane), a member of the order Gruiformes, this primer set may be applicable across a broader range of bird species, although further validation is required.

In conclusion, our findings revealed a unique genetic structure in the *CHD1* gene of owls characterized by a specific insertion of approximately 600 bp in intron 17 of *CHD1-W*. This owl-specific intronic insertion may represent an ancestral transposon event that occurred during the course of evolution; however, further studies are needed to confirm its origin.

## Figures and Tables

**Table 1:** Samples used for sex identification in this study.

Species	Sample No.	Sample ID	Sex	Confirmation of Sex
*Tyto alba* (Barn owl)	1	NMNS-TA230314	Female	-*
*Tyto longimembris* (Eastern grass owl)	1	NMNS-TL230315	Female	-
*Asio otus* (Long-eared owl)	2	NIES-4452A	Male	-
		KU-21-To	Female	-
*Asio flammeus* (Short-eared owl)	1	KU-21-Izm	Female	Necropsy
*Strix uralensis hondoensis* (Ural owl)	2	NIES-6358A	Male	Necropsy
		NIES-7003A	Female	Necropsy
*Strix uralensis japonica* (Yezo Ural owl)	2	NIES-6939A	Male	Necropsy
		NIES-7008A	Female	Necropsy
*Otus sunia* (Oriental scops owl)	1	NIES-5871A	Female	Necropsy
*Otus elegans interpositus* (Ryukyu scops owl)	2	NIES-3282A	Male	Necropsy
		NIES-7030A	Female	Necropsy
*Otus semitorques* (Japanese scops owl)	2	NIES-661A	Male	-
		KU-21-Ko	Female	-
*Otus lettia* (Collared scops owl)	2	NMNS-OL220826	Male	-
		NMNS-OL221126	Female	-
*Ninox japonica* (Northern boobook)	2	NIES-5917A	Male	Necropsy
		NIES-5916A	Female	-
*Athene brama* (Spotted owlet)	2	NMNS-ABr230314-4	Male	-
		NMNS-ABr230314-3	Female	-
*Bubo bubo* (Eurasian eagle-owl)	1	NIES-419A	Male	-
*Grus monacha* (Hooded crane)	2	KU-21-51	Male	Necropsy
		KU-21-21	Female	Necropsy

*-, not analyzed.

**Table 2:** Sequences of the primers used in this study.

Primer Name	Nucleotide Sequence (5′ to 3′)	Reference
2550F	GTTACTGATTCGTCTACGAGA	Fridolfsson and Ellergen, 1999 [13]
2718R	ATTGAAATGATCCAGTGCTTG	
2505F	GTAGCATTTAATACGTAGCAG	Designed in this study
2742R	ATACCATACCTCTGATCCTTC	

**Table 3:** *CHD1* gene sequences determined in this study.

Species	Gene	Accession No.	Length (bp)
*T. alba* (Barn owl)	*CHD1-Z*	LC841896	639
	*CHD1-W*	LC841909	1073
*T. longimembris* (Eastern grass owl)	*CHD1-Z*	LC841897	639
	*CHD1-W*	LC841910	1073
*A. otus* (Long-eared owl)	*CHD1-Z*	LC841884	646
	*CHD1-W*	LC841898	1057
*A. flammeus* (Short-eared owl)	*CHD1-Z*	LC841885	646
	*CHD1-W*	LC841899	1052
*S. uralensis hondoensis* (Ural owl)	*CHD1-Z*	LC841886	641
	*CHD1-W*	LC841900	1051
*S. uralensis japonica* (Yezo Ural owl)	*CHD1-Z*	LC841887	641
	*CHD1-W*	LC841901	1052
*O. sunia* (Oriental scops owl)	*CHD1-Z*	LC841888	643
	*CHD1-W*	LC841902	1058
*O. elegans interpositus* (Ryukyu scops owl)	*CHD1-Z*	LC841889	643
	*CHD1-W*	LC841903	1056
*O. semitorques* (Japanese scops owl)	*CHD1-Z*	LC841890	646
	*CHD1-W*	LC841904	1065
*O. lettia* (Collared scops owl)	*CHD1-Z*	LC841891	648
	*CHD1-W*	LC841905	1055
*N. japonica* (Northern boobook)	*CHD1-Z*	LC841892	649
	*CHD1-W*	LC841906	1051
*A. brama* (Spotted owlet)	*CHD1-Z*	LC841893	654
	*CHD1-W*	LC841907	1033
*B. bubo* (Eurasian eagle-owl)	*CHD1-Z*	LC841895	641
*G. monacha* (Hooded crane)	*CHD1-Z*	LC841834	637
	*CHD1-W*	LC841835	462

## Data Availability

Data are contained within the article or Supplementary Materials.

## Supplementary Materials

The following supporting information can be downloaded at: https://www.mdpi.com/article/10.3390/genes16060653/s1, Figure S1: Agarose gel electrophoretic patterns of the PCR products amplified using the primer set 2550F/2718R from four owl species; Figure S2: Agarose gel electrophoretic patterns of the PCR products amplified using the primer set 2550F/2718R from 13 owl species; Figure S3: Sequence alignment of the *CHD1* sequence at the binding site for the primer set 2550F/2718R in various owl species; Table S1: Species used in the phylogenetic analysis.

## References

[1] Ito H., Sudo-Yamaji A., Abe M., Murase T., Tsubota T. (2003). Sex identification by alternative polymerase chain reaction methods in Falconiformes. Zool. Sci..

[2] Lee J.C.I., Tsai L.C., Hwa P.Y., Chan C.L., Huang A., Chin S.C., Wang L.-C., Lin J.-T., Linacre A., Hsieh H.-M. (2010). A novel strategy for avian species and gender identification using the CHD gene. Mol. Cell. Probes.

[3] Griffiths R., Double M.C., Orr K., Dawson R.J.G. (1998). A DNA test to sex most birds. Mol. Ecol..

[4] Dawson D.A., dos Remedios N., Horsburgh G.J. (2016). A new marker based on the avian spindlin gene that is able to sex most birds, including species problematic to sex with CHD markers. Zoo Biol..

[5] Griffiths R. (2000). Sex identification in birds. Semin. Avian Exot. Pet Med..

[6] Morinha F., Cabral J.A., Bastos E. (2012). Molecular sexing of birds: A comparative review of polymerase chain reaction (PCR)-based methods. Theriogenology.

[7] Wang L.C., Chen C.T., Lee H.Y., Li S.H., Lir J.T., Chin S.C., Pu C.-E., Wang C.-H. (2007). Sexing a wider range of avian species based on two CHD1 introns with a unified reaction condition. Zoo Biol..

[8] Ong A.H.K., Vellayan S. (2008). An evaluation of CHD-specific primer sets for sex typing of birds from feathers. Zoo Biol..

[9] Ellegren H., Sheldon B.C. (1997). New tools for sex identification and the study of sex allocation in birds. Trends Ecol. Evol..

[10] Esaki M., Ito G., Tokorozaki K., Matsui T., Masatani T., Amano K., Ozawa M. (2022). Prevalence and organ tropism of crane-associated adenovirus 1 in cranes overwintering on the Izumi plain, Japan. Transbound. Emerg. Dis..

[11] Fridolfsson A.K., Ellegren H. (1999). A Simple and Universal Method for Molecular Sexing of Non-Ratite Birds. J. Avian Biol..

[12] Vucicevic M., Stevanov-Pavlovic M., Stevanovic J., Bosnjak J., Gajic B., Aleksic N., Stanimirovic Z. (2013). Sex Determination in 58 Bird Species and Evaluation of CHD Gene as a Universal Molecular Marker in Bird Sexing. Zoo Biol..

[13] Yuda P., Saputra A.W. (2021). Eggshell membrane for DNA sexing of the endangered Maleo (*Macrocephalon maleo*). F1000Research.

[14] Çakmak E., Akın Pekşen Ç., Bilgin C.C. (2017). Comparison of three different primer sets for sexing birds. J. Vet. Diagn. Investig..

[15] Hatakeyama H., Nakamura Y., Konaka T., Nishida S., Kriangwanich W., Uematsu K., Tsuchida S. (2020). Molecular sexing in Japanese murrelet (*Synthliboramphus wumizusume*) and a tandem-repeat polymorphism on the W chromosome. Sci. Rep..

[16] Brommer J.E., Karell P., Pihlaja T., Painter J.N., Primmer C.R., Pietiäinen H. (2003). Ural owl sex allocation and parental investment under poor food conditions. Oecologia.

[17] Hörnfeldt B., Hipkiss T., Fridolfsson A.K., Eklund U., Ellegren H. (2000). Sex ratio and fledging success of supplementary-fed Tengmalm’s owl broods. Mol. Ecol..

[18] Wang L.C., Severinghaus L.L., Chen C.T., Liu L.Y., Pan C.H., Huang D., Lee H.Y., Lir J.T., Chin S.C., Pu C. (2008). Sex identification of owls (family Strigidae) using oligonucleotide microarrays. J. Hered..

[19] Hebert P.D.N., Stoeckle M.Y., Zemlak T.S., Francis C.M. (2004). Identification of birds through DNA barcodes. PLoS Biol..

[20] Kumar S., Stecher G., Li M., Knyaz C., Tamura K. (2018). MEGA X: Molecular evolutionary genetics analysis across computing platforms. Mol. Biol. Evol..

[21] Kumar S., Stecher G., Tamura K. (2016). MEGA7: Molecular Evolutionary Genetics Analysis Version 7.0 for Bigger Datasets. Mol. Biol. Evol..

[22] Abdul-Rahman I.I., Awumbila B., Jeffcoate I.A., Robinson J.E., Obese F.Y. (2015). Sexing in Guinea fowls (*Numida meleagris*). Poult. Sci..

[23] Kaewhom P., Srikijkasemwat K. (2021). Efficacy of two primer sets used in the sex identification of rufous-winged buzzard (*Butastur liventer*). Open Vet. J..

[24] Wink M., El-Sayed A., Sauer-Gürth H., Gonzalez J. (2009). Molecular Phylogeny of Owls (Strigiformes) Inferred from DNA Sequences of the Mitochondrial Cytochrome b and the Nuclear RAG-1 gene. Ardea.

[25] Salter J., Oliveros C., Hosner P., Manthey J., Robbins M., Moyle R., Brumfield R., Faircloth B. (2020). Extensive paraphyly in the typical owl family (Strigidae). The Auk.

[26] Stehlíková Sovadinová S., Mekadim C., Korpimäki E., Mrázek J., Kouba M. (2024). Comparison three primer pairs for molecular sex determination in Eurasian pygmy owls (*Glaucidium passerinum*). Sci. Rep..

[27] Liu C.W., Hou H.Y., Hsieh H.I., Jang-Liaw N.H. (2024). Sex identification of birds in Taipei Zoo. Zoo Biol..

[28] Kulibaba R.O., Liashenko Y.V. (2021). Analysis of CHD Gene Polymorphism as a Model Object for Molecular Sexing of Eurasian Eagle-Owl (*Bubo bubo*). Cytol. Genet..

