# Supplementary material for: Molecular Sexing in Owls (Aves, Strigiformes) and the Unique Genetic Structure of the Chromodomain Helicase DNA-Binding Protein 1 (CHD1) Gene on Chromosome W
Source: Genes (Basel). 2025 May 28;16(6):653. doi: 10.3390/genes16060653 (PMC12191965; doi:10.3390/genes16060653)
Supplement: Supplementary file 1 [file genes-16-00653-s001.zip › Supplementary Figures.pdf]

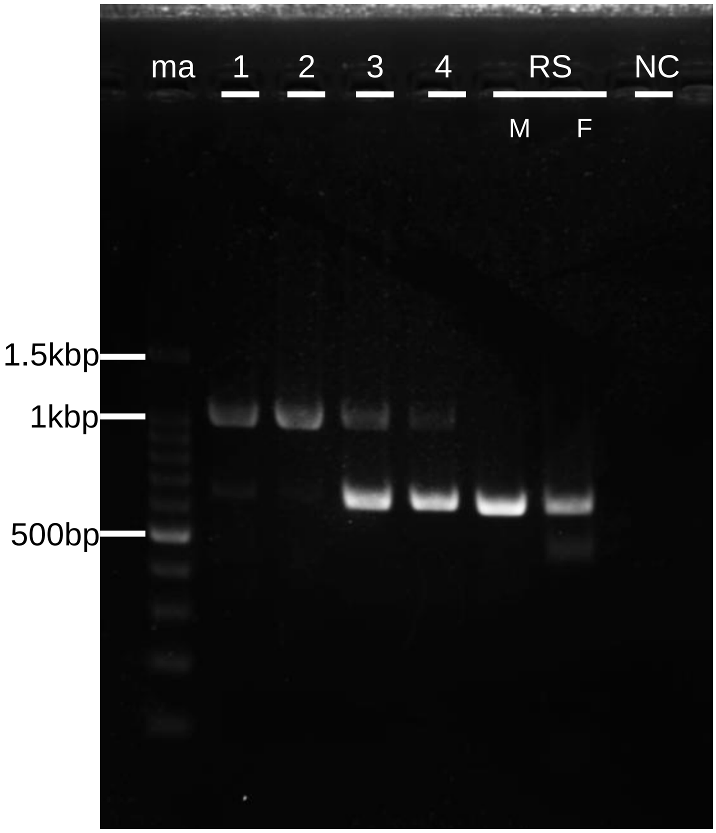

Supplementary Figure S1. Agarose gel electrophoretic patterns of the PCR products amplified using the primer set 2550F/2718R from four owl species. The previously reported primer set 2550F/2718R (Fridolfsson & Ellegren, 1999) was used for the molecular sexing of the four owls. 1: *Asio otus* (long-eared owl); 2: *Otus semitorques* (Japanese scops owl); 3: *Strix uralensis hondoensis* (Ural owl); 4: *Ninox japonica* (northern boobook); RS: *Grus monacha* (hooded crane) served as a reference sample; NC: nuclease-free water served as a negative control; M: Male; F: Female; ma: 100-bp DNA ladder used as a marker.

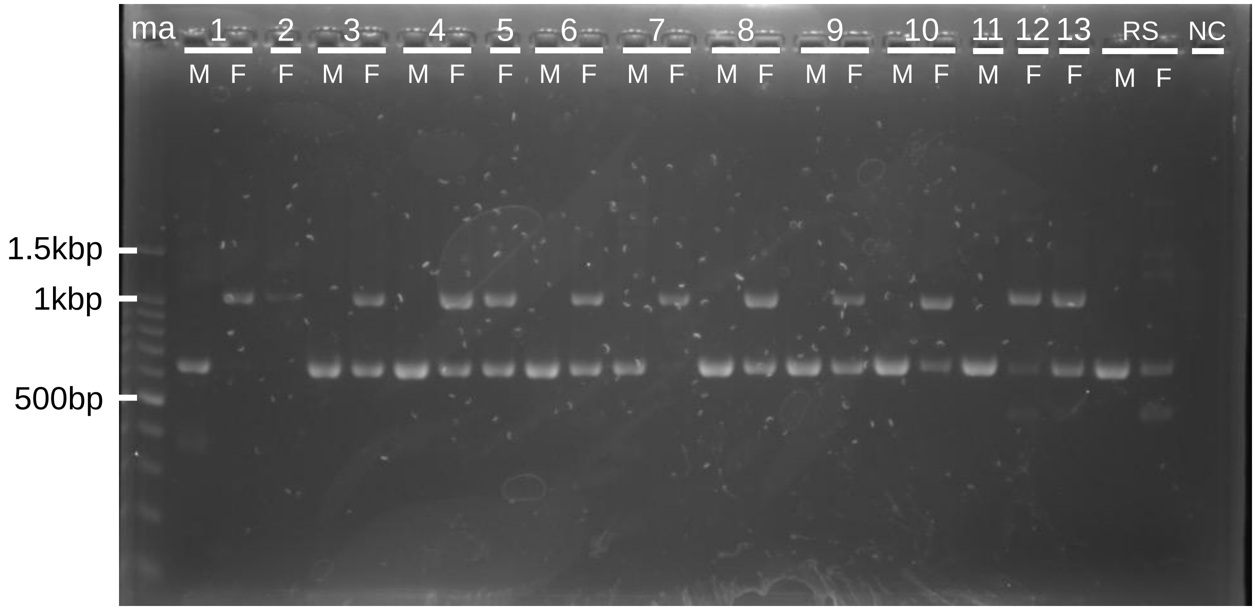

Supplementary Figure S2. Agarose gel electrophoretic patterns of the PCR products amplified using the primer set 2550F/2718R from 13 owl species. The previously reported primer set 2550F/2718R (Fridolfsson & Ellegren, 1999) was used to analyze 21 samples, including 9 males (M) and 12 females (F), from 13 owls. A *Grus monacha* (hooded crane) sample was included as a reference sample (RS), and nuclease-free water was used as a negative control (NC). . 1: *Asio otus* (long-eared owl), 2: *Asio flammeus* (short-eared owl), 3: *Strix uralensis hondoensis* (Ural owl), 4: *Strix uralensis japonica* (Yezo Ural owl), 5: *Otus sunia* (oriental scops owl), 6: *Otus elegans interpositus* (Ryukyu scops owl), 7: *Otus semitorques* (Japanese scops owl), 8: *Otus lettia* (collared scops owl), 9: *Ninox japonica* (northern boobook), 10: *Athene brama* (spotted owlet), 11: *Bubo bubo* (Eurasian eagle-owl), 12: *Tyto alba* (barn owl), 13: *Tyto longimembris* (eastern grass owl), ma: 100-bp DNA ladder used as a marker.

(A)

Primer 2550F	G T T A C T G A T T C G T C T A C G A G A
Hooded crane (KU 21-21) Female CHD1-W	. C . . . .
Long-eared owl (KU 21-To) Female CHD1-W	. C . . . .
Short-eared owl (KU 21-Izm) Female CHD1-W	. C . . . .
Ural owl (NIES 7003A) Female CHD1-W	. C . . . .
Yezo Ural owl (NIES 7008A) Female CHD1-W	. C . . . .
Oriental scops owl (NIES 5871A) Female CHD1-W	. C . . . .
Ryukyu scops owl (NIES 7030A) Female CHD1-W	. C . . . .
Japanese scops owl (KU 21-Ko) Female CHD1-W	. C . . . .
Collared scops owl (NMNS OL221126) Female CHD1-W	. C . . . .
Northern boobook (NIES 1019A) Female CHD1-W	. C . . . .
Spotted owlet (NMNS ABr230314-3) Female CHD1-W	. C . . . .
Barn owl (NMNS TA230314) Female CHD1-W	. C . . . . A . . . .
Eastern grass owl (NMNS TL230315) Female CHD1-W	. C . . . . A . . . .

(B)

Primer 2718R	C A A G C A C T G G A T C A T T T C A A T
Hooded crane (KU 21-52) Male CHD1-Z	. . . . T . . .
Hooded crane (KU 21-21) Female CHD1-Z	. . . . T . . .
Long-eared owl (NIES 4452A) Male CHD1-Z	. . . . T . . .
Long-eared owl (KU 21-To) Female CHD1-Z	. . . . T . . .
Short-eared owl (KU 21-Izm) Female CHD1-Z	. . . . T . . .
Ural owl (NIES 6358A) Male CHD1-Z	. . . . T . . .
Ural owl (NIES 7003A) Female CHD1-Z	. . . . T . . .
Yezo Ural owl (NIES 6939A) Male CHD1-Z	. . . . T . . .
Yezo Ural owl (NIES 7008A) Female CHD1-Z	. . . . T . . .
Oriental scops owl (NIES 5871A) Female CHD1-Z	. . . . T . . .
Ryukyu scops owl (NIES 3282A) Male CHD1-Z	. . . . T . . .
Ryukyu scops owl (NIES 7030A) Female CHD1-Z	. . . . T . . .
Japanese scops owl (NIES 661A) Male CHD1-Z	. . . . T . . .
Japanese scops owl (KU 21-Ko) Female CHD1-Z	. . . . T . . .
Collared scops owl (NMNS OL220826) Male CHD1-Z	. . . . T . . .
Collared scops owl (NMNS OL221126) Female CHD1-Z	. . . . T . . .
Northern boobook (NIES 5917A) Male CHD1-Z	. . . . T . . .
Northern boobook (NIES 1019A) Female CHD1-Z	. . . . T . . .
Spotted owlet (NMNS ABr230314-3) Female CHD1-Z	. . . . T . . .
Spotted owlet (NMNS ABr230314-4) Male CHD1-Z	. . . . T . . .
Eurasian eagle-owl (NIES 419A) Male CHD1-Z	. . . . T . . .
Barn owl (NMNS TA230314) Female CHD1-Z	. . . . T . . .
Eastern grass owl (NMNS TL230315) Female CHD1-Z	. . . . T . . .

(C)

Primer 2718R	C A A G C A C T G G A T C A T T T C A A T
Hooded crane (KU 21-21) Female CHD1-W	. . . . T . . .
Long-eared owl (KU 21-To) Female CHD1-W	. . . . - . . .
Short-eared owl (KU 21-Izm) Female CHD1-W	. . . . - . . .
Ural owl (NIES 7003A) Female CHD1-W	. . . . - . . .
Yezo Ural owl (NIES 7008A) Female CHD1-W	. . . . - . . .
Oriental scops owl (NIES 5871A) Female CHD1-W	. . . . - . . .
Ryukyu scops owl (NIES 7030A) Female CHD1-W	. . . . - . . .
Japanese scops owl (KU 21-Ko) Female CHD1-W	. . . . - . . .
Collared scops owl (NMNS OL221126) Female CHD1-W	. . . . - . . .
Northern boobook (NIES 1019A) Female CHD1-W	. . . . - . . .
Spotted owlet (NMNS ABr230314-3) Female CHD1-W	. . . . - . . .
Barn owl (NMNS TA230314) Female CHD1-W	. . . . - . . .
Eastern grass owl (NMNS TL230315) Female CHD1-W	. . . . - . . .

Supplementary Figure S3. Sequence alignment of the *CHD1* sequence at the binding site for the primer set 2550F/2718R in various owl species. A: *CHD1-W* sequence at the binding site for the primer 2550F, B: *CHD1-Z* sequence at the binding site for the primer 2718R, C: *CHD1-W* sequence at the binding site for the primer 2718R. A dot (.) indicates the same nucleotide as the primer sequence. A dash (-) indicates deletion of nucleotides.
