# Supplementary material for: Molecular Sexing in Owls (Aves, Strigiformes) and the Unique Genetic Structure of the Chromodomain Helicase DNA-Binding Protein 1 (CHD1) Gene on Chromosome W
Source: Genes (Basel). 2025 May 28;16(6):653. doi: 10.3390/genes16060653 (PMC12191965; doi:10.3390/genes16060653)
Supplement: Supplementary file 1 [file genes-16-00653-s001.zip › Supplementary Table S1.pdf]

Supplementary Table S1. Species used in the phylogenetic analysis.

Family	Species	Accession No.	
		CHD1-Z	*CHD-W*
Strigidae	*Asio flammeus*	LC841885*	LC841899*
	Asio otus	LC841884*	LC841898*
	Athene brama	LC841893*	LC841907*
	Aegolius acadicus	KF601362.1	N/A
	Aegolius funereus	KX516526.1	N/A
	Ninox japonica	LC841892*	LC841906*
	Otus semitorques	LC841890*	LC841904*
	Otus lettia	LC841891*	LC841905*
	Otus sunia	LC841888*	LC841902*
	Otus elegans	LC841889*	LC841903*
	Megascops asio	KF601364.1	HQ593874.1
	Bubo scandiacus	KF601366.1	N/A
	Bubo bubo	LC841895*	N/A
	Strix nebulosa	KF601354.1	N/A
	Strix uralensis japonica	LC841887*	LC841901*
	Strix uralensis hondoensis	LC841886*	LC841900*
Tytonidae	*Tyto alba*	LC841896*	LC841909*
	Tyto longimembris	LC841897*	LC841910*
Gruidae	*Grus monacha*	LC841834*	LC841835*
	Grus grus	EU814903.1	EU814910.1
	Grus japonensis	EF078970.1, EU814902.1	EU814909.1
	Grus virgo	EU814906.1	EU814913.1
	Grus antigone	KC676707.1	N/A
	Grus leucogeranus	EU814904.1	EU814911.1
	Balearica regulorum	EF078972.1, EU814907.1	EF078973.1, EU814914.1
Ciconiidae	*Ciconia boyciana*	EU814908.1	EU814915.1
Accipitridae	*Aquila chrysaetos*	HQ230022.1, AB112951.1	AB112943.1, HQ230023.1
	Nisaetus nipalensis	AB112958.1	AB112950.1
	Accipiter nisus	AB112953.1	AB112945.1
	Accipiter gentilis	AB112952.1	AB112944.1
	Milvus migrans	AB112957.1	AB112949.1

Megapodiidae	*Macrocephalon maleo*	MT074328.1, MT074329.1	MT074330.1
Alcidae	*Fratercula cirrhata*	AB597010.1	AB597009.1
Columbidae	*Streptopelia turtur*	LR594555.2	OU015480.1
Columbidae	*Columba livia*	AY517719.1	AY517718.1
Procellariidae	*Puffinus yelkouan*	MF662601.1	MF662602.1
	Calonectris diomedea	MF662597.1	MF662598.1
Hydrobatidae	*Hydrobates pelagicus*	MF662599.1	MF662600.1
Phalacrocoracidae	*Phalacrocorax capillatus*	AB080660.1	AB080661.1
Corvidae	*Corvus macrorhynchos*	AB246034.1	AB246036.1
	Corvus frugilegus	HM244402.1	HQ230021.1
	Corvus corone	AB246035.1	AB246037.1
Threskiornithidae	*Platalea minor*	AY464013.1	AY464014.1
Falconidae	*Falco peregrinus*	AB112955.1, KF601359.1	AB112947.1
	Falco tinnunculus	AB112956.1	AB112948.1
Psittacidae	*Ara militaris*	DQ331021.1	DQ331022.1
Caprimulgidae	*Caprimulgus europaeus*	OU015527.1	OU015537.1
Spheniscidae	*Pygoscelis papua*	GU451226.1	GU451230.1
	Aptenodytes patagonicus	GU451225.1	GU451229.1
	Spheniscus magellanicus	GU451227.1	GU451231.1
Threskiornithidae	*Threskiornis melanocephalus*	EF078968.1	EF078969.1
Apodidae	*Aerodramus inexpectatus*	MN819581.1	MN819580.1

*-, sequenced in this study; N/A, not applicable
